# Hospitals and Their Special Needs

**Published:** 1920-06-26

**Authors:** 


### HOSPITALS AND THEIR SPECIAL NEEDS.

**ALL SAINTS' CONVALESCENT HOSPITAL, EASTBOURNE.**—The Hospital, after being occupied for nearly three years as a military hospital for the Canadian troops, was reopened for the full reception of men, women, and children convalescent patients last month, with the same accommodation as before the war. Previous to the reopening it was found necessary to entirely re-roof the building and to renew all the drainage, the cost of which ran into thousands of pounds An urgent appeal is made to the charitable public to contribute.

**BELGRAVE HOSPITAL FOR CHILDREN, CLAPHAM ROAD,** like all other hospitals, is faced with a largely increased cost of maintenance. In 1914 £4,400 was spent, whilst the expenditure for this year is estimated at a figure approaching £8,500. During the war paintings and repairs got in arrears, and a considerable sum is required to overtake the back work and keep the premises in a proper state of repair. The matron is short of probationers, and she will be pleased to supply all particulars to those anxious to take up nursing. The scale of salaries has been revised, the staff augmented, and the hours of duty considerably reduced. Thos. Clapham, Secretary.

**BOLINGBROKE HOSPITAL, WANDSWORTH COMMON, S.W. 11,** serves a population of 750,000 inhabitants. Its special needs are reduction of the debt, which is now £7,000, and a new *x*-rays apparatus. The hospital was the first in London to use *x*-rays, as the early experiments of the late Dr. Lyster were first carried out here.

**CHELSEA HOSPITAL FOR WOMEN** makes an urgent appeal on behalf of those on its "waiting-list," whose admission is greatly delayed for want of accommodation. As soon as it can raise £20,000 and complete with £7,000 in hand the provision for a nurses' home, the remaining half of the hospital wards will be freed for patients. It is calculated that, pending that provision, 700 patients annually are spending months of pain which they might otherwise be saved. Everything possible is being done at this hospital to preserve the best effects of the voluntary system.

**CITY OF LONDON MATERNITY HOSPITAL, CITY ROAD, E.**—Since its establishment in 1750 the hospital has delivered 79,556 women of 80,440 children. Last year 428 women were attended in their own homes. It caters for the poorest, many of whom cannot afford even the smallest donation. The hospital has a consultative maternity centre, and an ante-natal clinic and child-welfare centre is held twice weekly. Pupil-midwives and nurses are trained, and medical students admitted to the practice of the hospital. Its great need is a laundry on the premises and a convalescent home outside London. The latter would enable some hundreds more patients to be admitted annually. Its debt now stands at £6,800.

**CHISLEHURST, ORPINGTON, AND CRAY VALLEY HOSPITAL, ST. PAUL'S CRAY, KENT.**—The hospital is at present in debt to our bankers to a considerable extent, owing to the increased cost of feeding the patients and staff, and some necessary repairs and painting must be undertaken in order to preserve the fabric and woodwork.

**CITY OF LONDON HOSPITAL FOR DISEASES OF THE CHEST, VICTORIA PARK, E. 2.**—The large increase in the cost of maintaining the hospital, estimated at £32,000 for the present year, compels the Committee to plead with all to whom the voluntary system of hospitals is precious to assist them in some measure. There are structural improvements and additions which were curtailed on the outbreak of the war now urgently necessary, but until the general requirements are assured the Committee cannot undertake them. Additional facilities for research work have lately been provided. The hospital has deserved well of the public by reason of its valuable co-operation in respect of needs arising out of the war. Contributions will be gratefully received by the Treasurer, Sir G. Wyatt Truscott.

**DREADNOUGHT SEAMEN'S HOSPITAL, GREENWICH.**—Thanks to the generous support accorded to the Seamen's Hospital Society by those who realise what we owe to the sailor, the Society is able to show a small balance on the right side. This has not been achieved without a great deal of care and hard work. Figures speak for themselves: the income from investments is only £7,480, while the expenditure amounts to £49,000. Unfortunately and unavoidably the cost of maintenance is still going up, and it is only by the continued support of all those who are aware of what the sailor has done, and is doing, for this country that the charity can hope to keep abreast of the tide.

**EAST LONDON HOSPITAL FOR CHILDREN.**—The present position is much the same as most hospitals. The deficit in last year's account was £1,830. The debt to the bank on December 31, 1919, was £2,200. At the present time the hospital owes its bankers £5,350, with the prospect of being much increased before the end of the year. A much larger expenditure than last year must be faced, as certain repairs most necessary for the upkeep of the buildings and held in abeyance for many reasons during the war must be carried out.

**EVELINA HOSPITAL FOR SICK CHILDREN, SOUTHWARK BRIDGE ROAD, S.E.**—Although the "Evelina" is the only large children's hospital for the whole of South London, and is situated in its most destitute district, its financial position, in the words of Viscount Duncannon, the President, at the recent annual meeting, is "really desperate." It is, therefore, not only important, but essential, that a good response shall be forthcoming to the urgent appeal now being made to raise a special sum of £10,000 (additional to the ordinary income).

**GROSVENOR HOSPITAL FOR WOMEN, VINCENT SQUARE, WESTMINSTER, S.W. 1.**—The principal need of this hospital, like all others, is money to carry on. With the continued rise in the price of every necessary, the annual expenditure ever increases. The bank overdraft to the end of April was £1,000, and the financial position is causing great anxiety. W. J. Davidson, Secretary.

**GREAT NORTHERN CENTRAL HOSPITAL.**—A special appeal has been issued for £10,000 to meet the present very urgent needs. The hospital was established in 1856 in Caledonian Road as a small hospital with an

**Hospitals and their Special Needs**—(*continued*).

out-patient department, now a great institution with 310 beds, namely, 220 at its headquarters in the Holloway Road, for the most urgent cases, sixty in the "Summerlee" Hospital of Recovery at Finchley, to assist in quick restoration of health, and thirty in the Convalescent Home at Clacton-on-Sea. Unless more substantial help is obtained this year the Maintenance Fund will be from £12,000 to £14,000 short, and this will be added to the deficiency of £14,000 last year. Rev. J. Ough, M.A., Chaplain of the Great Northern Central Hospital, is preaching on June 27 on behalf of the Hospital Sunday Fund, at St. Peter's, Hornsey, in the morning, and at St. James', Holloway, in the evening.

**GUY'S HOSPITAL, S.E. 1.**—In common with other charities, great and small, "Guy's" is compelled to ask for further and immediate help. The income of the old endowment (£30,000) is now less than one-quarter of the total ordinary expenditure; and a large part of the difference has to be provided every year by the benefactions of the public. It cannot be believed that help will be refused and that there will be lacking to-day the spirit of compassion which, in former times, moved the citizens of London to give freely of their possessions for the relief of suffering. Treasurer, Viscount Goschen; Bankers, the Bank of England.

**HAMPSTEAD GENERAL AND NORTH-WEST LONDON HOSPITAL, HAVERSTOCK HILL, N.W. 3.**—The Hospital serves the North-West London district, including the populous area of Camden and Kentish Town. The number of patients daily under treatment in the wards at Haverstock Hill averages 112, and many out-patients are dealt with in the well-equipped department situated at Camden Town. It is estimated that a sum possibly exceeding £22,000 will be required to meet the total expenditure incurred during the current year. The debt at this date exceeds £5,000. Mr. Harold Wigg is the Secretary.

**HOME HOSPITAL FOR WOMEN, 187 HIGH STREET, STOKE NEWINGTON, N. 16.**—There is accommodation for twenty-five patients, and in addition to taking the ordinary cases, the institution meets the needs of those who require a certain amount of surgical and medical care, but are not ill enough for admission to the general hospitals; maternity cases are also taken. Like all others, this hospital is heavily in debt owing to the increased cost of maintenance, and subscriptions are earnestly requested.

**HOSPITAL AND HOME FOR INCURABLE CHILDREN, NORTHCOURT, 30 COLLEGE CRESCENT, HAMPSTEAD, N.W.**—The name of the hospital has been changed, as the Committee of Management thought that the word "incurable" had a depressing influence on the children brought to the hospital, and also because in many cases cure has been effected. Funds are urgently needed, as apart from the £4,400 mortgage on the hospital, there is an overdraft from the bankers for £940. Hon. Secretary, Mr. Edward Brown.

**HOSPITAL FOR EPILEPSY AND PARALYSIS, MAIDA VALE, W.,** has not been in debt for over forty years, and is now in urgent need of £3,000 to pay bills, £2,000 for painting and decoration delayed during the war, £10,000 for the extension of the out-patient department, and £10,000 for the treatment of patients, making a total of £25,000. In addition to this the hospital has plans for a very important research department, and it is estimated that a ten years' research into the causes and treatment of nervous diseases will be an expense of a further £25,000. Contributions may be sent to Mr. H. W. Burleigh, the Secretary.

**HOSPITAL FOR SICK CHILDREN, GREAT ORMOND STREET, W.C. 1.**—The fact that the Committee owe the hospital bankers at the present moment £15,000, which it is anticipated will be increased to £20,000 by the end of July, shows that the financial position needs alleviation. Acting Secretary, Mr. James McKay.

**KING'S COLLEGE HOSPITAL,** with its 400 beds, large casualty department, medical and surgical, extensive out-patient department, as well as special departments, cardiological, radiological, physio-therapeutic, neurological, urological, psychological, orthopædic, ante-natal, and maternity departments, is the Great South London Hospital, ministering not only to the needs of a vast population in South London, but is constantly rendering assistance in cases of emergency from all parts of the country. It will cost at least £100,000 a year to run the hospital efficiently and continue its great work for the sick and suffering. Towards this enormous annual expenditure not more than about £40,000 a year income is assured, thus an additional £60,000 a year must be raised if the open door is to be maintained.—R. J. Coles, Secretary.

**LONDON FEVER HOSPITAL.**—The above hospital is not rate-supported, and was founded in 1802 by the inhabitants of London for the prevention and cure of infectious fevers. It is, therefore, the parent hospital in London for infectious diseases, and is now in danger of having to close its doors for want of funds. Over 100,000 patients have been treated since 1802, and nearly 1,000 yearly for the past three years. There are 178 beds, and patients pay an amount towards their cost, but the expenditure absolutely necessary for efficient work far exceeds the receipts, and the committee have been compelled to borrow money from their bankers, and there is now a very considerable overdraft. Immediate help is very urgently needed. The Secretary is Commander T. J. Farrell, D.S.C.

**LONDON HOSPITAL, WHITECHAPEL, E.**—One word—"Desperate"—sums up the present position of England's biggest medical charity—the London Hospital. It is, indeed, in very real peril of closing—of actual closing for lack of funds, not of threatened closing to lure them. The financial positions stands thus: with a certain income of £36,000 a year the hospital has to meet essential expenditure amounting to £200,000 a year. It owes £85,000. Every security is pledged. It can borrow no more. It is the blackest hour in the whole 180 years of the hospital's history.

**MATERNITY, CHARITY, AND DISTRICT NURSES' HOME, HOWARDS ROAD, PLAISTOW, E.**—Owing to the increasing need for more accommodation for maternity cases in their temporary hospital, the Charity have decided to build a new maternity hospital at a cost of about £25,000. During the year 1919 the Charity's staff attended to more than one-half the total number of births in the districts of

**Hospitals and their Special Needs**—(*continued*).

West Ham and East Ham. Donations towards the building fund are asked for. The Secretary is Mr. Chas. H. Andrews.

**MARY YOLLAND HOME FOR INCURABLE GIRLS (INTERMEDIATE) AT UPPER HALE, FARNHAM,** is greatly in need of funds to meet the heavy expenses of increased salaries and wages, food, fuel, and drugs; also for necessary repairs to buildings and furniture. The debt on the land has been cleared off, but some more rooms are urgently needed. Miss M. Yolland, Hon. Treasurer.

**MEMORIAL HOSPITAL, MILDMAY PARK, N.,** is situated in a quiet and ideal spot for the sick. Difficulties in administration are great, and funds are sadly needed. Repairs are necessary, for which a large sum is required, an entirely new roof being set down by an expert as an absolute necessity. Furthermore, in case of fire, there is no extra means of escape but rope ladders from the wards, the old iron escape being completely worn out. At the present high cost of living it is impossible to pay for such things out of ordinary income; we therefore appeal to the public for help to supply these great needs. Nurses will find that at this Hospital, though small, a very sound training may be obtained; the work is good and varied, and much knowledge is to be gained. A charming nurses' home stands apart from the hospital in the same grounds. Subscriptions and donations will be gratefully received by the Secretary.

**METROPOLITAN HOSPITAL, KINGSLAND ROAD, E. 8.**—Present financial position: Tradesmen's accounts outstanding, £19,000; mortgage, £10,000. Special needs: Suitable accommodation for nurses, and renovation of kitchens, cooking appliances, stores, etc. The hospital has very few investments, and the definite income generally is scanty. Mr. Guy B. Dale is the Secretary.

**MIDDLESEX HOSPITAL.**—The Hospital has a very special need at the present time—namely, the improvement and extension of its out-patient department. This urgent work has been in view for a long time, but the war necessarily checked any active steps towards a start. But it is now hoped that support will be forthcoming to justify a definite movement. The available space for the reception and treatment of out-patients is utterly inadequate to do justice to their greatly increased and increasing numbers, particularly in view of the special clinics which have come into being during the last few years. Under the present conditions of the labour and material market the sum of £80,000 to £100,000 is required to carry out the projected improvements. Contributions should be addressed to the Earl of Athlone, or to the Secretary-Superintendent, Mr. Walter Kewley.

**MILDMAY MISSION HOSPITAL, AUSTIN STREET, E. 2.**—The great aim and object of the Hospital is the fulfilment of our Lord's injunction, "Heal the sick . . . and say unto them, the Kingdom of God is come nigh unto you." It has been invaluable help to Missionary Societies who have used it as a training-ground for their students. Fifty doctors and twenty-four qualified nurses have gone from the hospital to work in the foreign field, and 213 women missionaries have received training in the out-patient departments for work abroad. It serves particularly the districts of Bethnal Green, Shoreditch, and Hoxton, and receives also patients from all parts of London and the country. It treats annually about 650 in-patients, and the out-patients' attendances number over 30,000.

**MILLER GENERAL HOSPITAL FOR SOUTH-EAST LONDON, GREENWICH ROAD, S.E. 10.**—It is anticipated that for the maintenance of the hospital and the convalescent home at Bexhill will cost at least £22,000 this year, but the assured income from investments is only £925. There are only seventy-six beds, and the out-patient attendances are more than one-third of the number at the London Hospital, which shows the great need for a proposed extension. The beds are naturally always full, and hundreds of very serious cases have to be turned away through lack of accommodation, quite apart from the long list of patients who are always on the waiting list from the regular out-patient departments.

**MOUNT VERNON HOSPITAL, NORTHWOOD, MIDDLESEX.**—Additional support is needed. The cost of maintenance has increased owing to the high cost of all hospital requirements. At the moment the Committee owe their bankers £6,000, in addition to which £2,000 stock has been realised to meet expenditure. A continuance of the present rate of expenditure means a further deficit at the end of the year, unless greater support can be obtained. The new block for children, begun in 1913—on which work was suspended in 1914—remains unfinished. £30,000 will now be required for its completion. Secretary, Mr. H. J. Morton.

**NATIONAL HOSPITAL FOR THE PARALYSED AND EPILEPTIC, QUEEN SQUARE, W.C.**—The financial position is so acute that the whole of the 170 beds in the main hospital have had to be closed, not for long, it is hoped. The hospital continues its large out-patient department, its convalescent home at East Finchley, and the separate hospitals for discharged Service men, maintained by the Ministry of Pensions, at Clapham Park and Bloomsbury. The Earl of Harrowby is the Treasurer.

**NELSON HOSPITAL, MERTON** (formerly the S. Wimbledon and Merton Cottage Hospital).—This hospital was founded in 1900, in an old house in Merton Road, Wimbledon. In 1905 advantage was taken of the Trafalgar Centenary celebrations to start a fund for providing an up-to-date hospital on a central site, and to associate with it the name of Nelson, who lived at Merton for three years and intended to spend the remainder of his life there after retirement from active service. Thanks to generous support, the present hospital was erected and opened, free of debt, in 1911. Funds for maintenance are greatly needed.

**PADDINGTON GREEN CHILDREN'S HOSPITAL.**—The hospital has forty-six beds and a convalescent home at Slough with twenty-four beds. Ordinary receipts, £7,237; ordinary expenditure, £8,896. Seven hundred and ninety-six in-patients were admitted during 1919, and 11,220 out-patients (new cases); 41,300 out-patient attendances were recorded. Total debt to-day, £2,500. F. Stanley Cheer, Secretary.

**PASSMORE EDWARDS HOSPITAL FOR WILLESDEN, HARLESDEN ROAD, N.W.**—The Passmore Edwards Hospital, Willesden, formerly known as the Cottage Hospital, has a scheme of extension to provide a

**Hospitals and their Special Needs**—(*continued*).

hospital of 120 beds on a freehold site already paid for adjoining the present hospital, utilising the present buildings for out-patients and offices, etc. This scheme has been publicly adopted as Willesden's War Memorial. All those who are interested in Willesden and its welfare are earnestly asked to send donations to the Secretary, Mr. H. Edgell Brown, at the Hospital, Harlesden Road, N.W. 10.

**PHILLIPS MEMORIAL HOMŒOPATHIC HOSPITAL AND DISPENSARY, BROMLEY, KENT.**—The needs of this institution include table linen of all kinds, cotton-wool dressings, and bandages. The present difficulty is a dwindling of annual subscriptions and an overdraft at the bank at present of about £50. Hon. Secretary, Mr. T. H. Cole.

**POPLAR HOSPITAL FOR ACCIDENTS, EAST INDIA DOCK ROAD, POPLAR, E. 14.**—The hospital has never been in debt. The policy is to do the work with absolute efficiency within the means provided by friends, and though it is desired to make it better by means of improvements, it may fairly be claimed that to-day the hospital is run as efficiently and sympathetically as any hospital in London. All expenditure has been increased by at least 100 per cent. Subscriptions or donations should, if possible, be increased accordingly. The Secretary is Mr. Percy Rogers.

**PRINCE OF WALES'S GENERAL HOSPITAL, TOTTENHAM.**—This is the only hospital in a district containing a population of 800,000 people. It contains 125 beds, is supported entirely by voluntary contribution, and requires an annual income of £18,000. Over 30,000 patients are treated each year, and the hospital is £10,000 in debt.

**QUEEN CHARLOTTE'S HOSPITAL.**—The demands for the services of the hospital are greater than ever before. Every year 2,000 patients are admitted to the wards, and 3,000 others are attended and nursed in their own homes. In the ante-natal and child-welfare departments over 5,000 patients are attended annually. There is a deficiency of over £11,000 on the income and expenditure account, which has accumulated during the past five years. The hospital had to postpone the extension which was about to be commenced on the outbreak of war, but the Committee are anxious now to proceed with a number of urgent improvements. They are, however, unable to do much without a considerable sum of money. It is estimated that quite £100,000 will be required. The Committee earnestly appeal for generous help to enable them to pay off the debt and to carry out the extensions and improvements which are so greatly needed. Contributions may be sent to the Secretary (Mr. Arthur Watts), at the Hospital, Marylebone Road, N.W. 1.

**QUEEN'S HOSPITAL FOR CHILDREN** will be compelled to close two wards, containing sixty-two beds, within three months, unless funds can be obtained to pay off the deficit. Some 800 little invalids annually receive every possible attention in these two wards. Failing additional support, there can be no alternative to this drastic step, and the Committee earnestly plead for help to avoid the threatened calamity. Secretary, T. Glenton-Kerr, Hackney Road, Bethnal Green, E. 2.

**ROYAL FREE HOSPITAL, GRAY'S INN ROAD, W.C. 1.**—The financial position of this hospital is causing grave anxiety. The expenditure during 1919 exceeded the income by no less than £20,371, and there is no doubt that a deficit to a similar extent will have to be faced during the present year. It is proposed to provide a limited number of beds upon a contributory basis, but this cannot materially reduce the very heavy responsibilities of the Board of Management.

**ROYAL LONDON OPHTHALMIC HOSPITAL (MOORFIELDS EYE HOSPITAL), CITY ROAD, E.C. 1.**—Owing to increased cost, the hospital, which for 116 years has been able to pay its way, is now quite unable to meet its expenditure. It is over £12,000 in debt to its bankers and tradesmen. It has had to sell £8,000 of its capital at great loss, merely to meet the ordinary requirements of maintenance. Unless ample funds are forthcoming, the only alternative is the closing down of a large part of the hospital. Robert J. Bland, Secretary.

**ROYAL MINERAL WATER HOSPITAL, BATH.**—The demands for admission are very great. A considerable proportion of the patients come from London, and it is to London that the Treasurer looks for increased support if the full number of beds is to be maintained. A scheme for the development of research into rheumatism, arthritis, and other diseases treated at Bath is being developed by the Committee. T. Kirby, Registrar.

**ROYAL NATIONAL HOSPITAL FOR CONSUMPTION, VENTNOR.**—Fifty years have passed since the first patient was received for treatment at the Ventnor Hospital, and at the end of 1919 the number of cases received had reached 30,830. The year 1919 was a record year in more ways than one. Firstly, the number of patients treated in the twelve months exceeded that of any preceding year by nearly 100. Secondly, the annual subscriptions reached a higher total than ever before; but, thirdly and lastly, expenditure exceeded income by £2,224.

**ROYAL NATIONAL SANATORIUM FOR CONSUMPTION AND DISEASES OF THE CHEST, BOURNEMOUTH.**—The need for a substantial increase in voluntary contributions is particularly urgent, owing to the deficit in the income for the past year, to a considerable extra expenditure which must be met this year on painting and other work which has accumulated during the war and can no longer be postponed, and to the continued increase in the prices of many commodities. In these circumstances of real and urgent need the Committee earnestly appeal for generous assistance.

**ROYAL WATERLOO HOSPITAL FOR CHILDREN AND WOMEN.**—The amount at present on loan at the bank is £4,250 to meet the current expenses of the hospital. In addition, the old nurses' home and isolation wards, which were to have been rebuilt when the war broke out, have now been condemned, and building operations which have the approval of the King Edward's Hospital Fund have been authorised immediately. The total cost is estimated at about £70,000, of which some £18,000 has already been raised. The Appeal Committee has recently been reformed, and is in urgent need of friends and helpers. Further information will gladly be given by applying to the Secretary, Mr. Alexander Pym, at the hospital.

(Continued on page 347.)

**Hospitals and their Special Needs**—(*continued*).

**ROYAL WESTMINSTER OPHTHALMIC HOSPITAL, KING WILLIAM STREET, STRAND, W.C. 2.**—Founded in 1816, this hospital has carried on more than a century of useful work, and yearly treats about 700 in-patients and deals with 33,000 attendances of out-patients. Last year the expenditure exceeded the income by about £1,300.

**ST. ANDREW'S HOSPITAL, DOLLIS HILL, LONDON, N.W. 2.**—Help is needed for the maintenance of the hospital; the paying off of the deficit of £1,005 on the account for 1919; the extinction during 1920 of the debt of £3,260 on the buildings; and the completion of the building.

**ST. ANDREW'S HOSPITAL, CLEWER.**—The expenditure has rapidly gone up from £3,000 to £6,000 a year, but the income has decreased. By strict economy and the raising of the rates for paying patients some headway is being made, but outside help is becoming more and more essential. Gifts in kind, such as bedding, blankets, crockery, and the like, will be gratefully received. A heavy outlay has been caused by the wearing out of the boiler and the steam apparatus.

**ST. BARTHOLOMEW'S HOSPITAL, WEST SMITHFIELD, E.C.**—In round figures £150,000 is required annually for the maintenance of this our oldest hospital, only about half of which comes from invested funds. A new block, including a nurses' home, is projected, and a separate fund is being raised for this purpose. The Treasurer is Viscount Sandhurst.

**ST. COLUMBA'S HOSPITAL, OR HOME OF PEACE.**—The hospital is not intended for chronic invalids, but for dying patients, for whom no provision is made in the general hospitals. Since the war the expenses have nearly doubled, and the contributions have not kept pace. The council have already borrowed £3,000, and their bankers have notified them that they cannot increase their advance. They are actually without means at the present time to meet current expenses. Mr. Arthur R. Godrich is the Secretary.

**ST. GEORGE'S HOSPITAL, S.W. 1.**—This Hospital, which was established 187 years ago, is still carrying on the work then commenced for the sick poor, as may be seen from the fact that in 1919 4,969 in-patients and 36,356 out-patients received treatment. The ordinary expenditure exceeded the ordinary income in 1919 by £33,311, and there seems every likelihood of a similar deficit this year, so that the kind assistance of all who may have the welfare of this hospital at heart is earnestly desired. There is now an overdraft at the bankers of £4,380. The Secretary is Mr. James M. Churchfield.

**ST. JOHN'S HOSPITAL FOR DISEASES OF THE SKIN, LEICESTER SQUARE, W.C. 2.**—It has always been a principle of this hospital to encourage patients to contribute to the cost of treatment as far as lies within their means, and it has been a cause of gratification to the board that they have thus been able to relieve the benevolent public to the extent of the sums so contributed. But charitable contributions are none the less necessary to the extent of £2,000 to £3,000 every year. Mr. George A. Arnaudin is the Secretary.

**ST. JOHN'S HOSPITAL, LEWISHAM.**—This Hospital receive financial support because, with the exception of the South Eastern Hospital for Children at Sydenham, it is the only voluntary hospital in the large Borough of Lewisham. The expenditure exceeded last year the income by £693 7s. 5d. The hospital has practically no endowment, and depends for its existence on donations and subscriptions.

**ST. LUKE'S HOSPITAL FOR ADVANCED CASES (FOUNDED IN 1893 AS ST. LUKE'S HOUSE, A HOME FOR THE DYING), 14 PEMBRIDGE SQUARE, BAYSWATER, W. 2.**—For cases of mortal illness in its latest phase. To provide skilled nursing and medical attendance, with the comforts of home. Admission free, irrespective of religious denomination. In addition to subscriptions and donations, which are required for the maintenance of the hospital, funds are urgently needed for the building fund, which at present amounts to only £6,682. The lease of the present premises, which are quite insufficient, will expire in 1923. Miss Helen E. Don is the Hon. Secretary.

**ST. MARY'S CONVALESCENT HOME, BIRCHINGTON-ON-SEA,** is in need of help at this moment on account of necessary repairs and painting, which could not be executed during wartime, but which have now been undertaken. Funds for maintenance are also required. The Hon. Lady Fremantle, 10 Draycott Place, S.W. 3, will be glad to receive contributions.

**ST. THOMAS'S HOSPITAL.**—The Governors of St. Thomas's Hospital are faced with very serious financial difficulties. At the beginning of the year £105,739 was owing to the bankers, in addition to liabilities to tradesmen and others, on December 31 last of £22,572. St. Thomas's has an endowment which in pre-war days enabled it, with the help received from its own supporters and friends, to carry on the hospital work and develop new departments as need arose. Prices have so greatly increased that the expenses can be no longer met as of old. The assured income from the endowment of the hospital in 1919 amounted to £67,376, and no increase on this amount can be looked for. The circumstances of every individual who is admitted to the wards or treated as an out-patient are inquired into by the Lady Almoners, and wherever possible contributions to meet the cost of the treatment of the patient are secured. It is, however, found that there is a very large proportion of the patients under treatment who do not benefit materially by the high wages now ruling. It is quite impossible to secure from this source anything like a sufficient sum to make up the deficit in the income. An appeal is therefore most earnestly made to all who can afford it to give every assistance in their power to enable this hospital, which is one of the oldest charities in London, to carry on its work as fully and as efficiently as in pre-war days. Annual subscriptions, donations, and legacies are particularly welcomed by the Governors. The total cost of maintenance and administration in 1919 was £154,472, whilst the total income amounted only to £111,953. The outlook for the present year is very serious unless the help which is so greatly needed is forthcoming.

**SAMARITAN FREE HOSPITAL FOR WOMEN, MARYLEBONE ROAD, N.W. 1.**—Princess Arthur of Connaught appeals for this hospital, of which she is President. It is desperately in need of funds, last year having closed with a deficit of £2,500. There is no possibility of the expenses being reduced; more

**Hospitals and their Special Needs**—(*continued*).

subscriptions must be obtained to carry on the work. The Princess knows the work personally, not only as President, but as having worked herself in the operating theatre and wards since May 1919, and can testify to the splendid results obtained. The money given gives life and health to hundreds of poor women every year. £5,000 to pay off the debt of £2,500, and to have the same amount to relieve anxiety as to the future, is asked for.

**SANTA CLAUS HOME, CHOLMELEY PARK, HIGHGATE, LONDON, N.**—This Surgical Nursing Home for poor children is in urgent need of funds, owing to the greatly increased cost of maintenance. Children with hip and spinal diseases, and other surgical cases needing skilled nursing, long rest, and care, are taken. During the twenty-nine years the home has been open the results have been most satisfactory, the majority of the children returning to their own homes fit for school or work. Donations to be sent to Miss Charles at the home.

**TEDDINGTON AND HAMPTON WICK COTTAGE HOSPITAL.**—Founded in 1875 and has twenty-four beds. Supported entirely by voluntary contributions, and, except for the services of the matron, and, of course, the staff, all the work that is done for the hospital, including that of the medical staff, is voluntary. The present hospital has really outgrown the needs of the present day, and the medical staff have for some time past unanimously been of opinion that a new hospital is needed. Honorary Secretary and Treasurer, Mr. F. Hugh Munby.

**VICTORIA HOSPITAL, CHELSEA.**—Financially the year 1919 was a very difficult one, as the total income only amounted to £10,685, as compared with £13,551 in 1918, and the expenditure increased from £11,078 in 1918 to £14,349 in 1919. To meet this the Committee had to sell some of the hospital investments at great loss, and, for the first time for many years, there was a deficit at the end of the year amounting to £3,663. The proposed improvements in the heating system and the enlargement of the nurses' home, although urgently needed, have had to be postponed owing to lack of funds.

**WESTMINSTER HOSPITAL.**—The income in 1919 fell short of the expenditure by £14,000. Efforts are being made to obtain contributions from patients, but these will not provide all that is needed.

